# Stress Distribution in Interlocking and Blocking Screw Fixation for Distal Tibial Intramedullary Nailing: A Finite Element Analysis

**DOI:** 10.3390/jcm14134769

**Published:** 2025-07-05

**Authors:** Gu-Hee Jung, Se-Lin Jeong, Jungtae Ahn

**Affiliations:** 1 Department of Orthopaedic Surgery, Gyeongsang National University, College of Medicine, Gyeongsang National University Changwon Hospital, Samjeongja-ro 1, Seongsan-gu, Changwon-si 51472, Republic of Korea; jyujin2001@hotmail.com; 2 Institute of Health Sciences (Medical ICT Convergence Research Center), College of Medicine, Gyeongsang National University, Jinju 52727, Republic of Korea; sl960826@naver.com

**Keywords:** distal tibial fracture, intramedullary nailing, blocking screws, finite element analysis, biomechanics

## Abstract

**Background/Objectives:** This study evaluates the structural strength of various fixation models for distal tibial fractures using finite element analysis, comparing the biomechanical performance of different interlocking and blocking screw configurations in intramedullary nail fixation. **Methods**: Finite element models were developed for three different screw configurations: two interlocking screws (T2S), three interlocking screws (T3S), and two interlocking screws combined with two blocking screws (T2SBS2). Material properties were assigned using SAWBONES^®^ models, reflecting established biomechanical characteristics. The models were subjected to static axial loading (800 Nm), internal rotation (3500 Nm), and external rotation stresses. The stress distribution and structural integrity of each fixation model were analyzed. **Results**: The T3S model demonstrated the lowest maximum von Mises stress values across all loading conditions. Under axial loading, the maximum VMS at the medial first screw contact was the lowest in the T3S model (65.96 MPa) compared to T2S (135.68 MPa) and T2SBS2 (150.30 MPa). Similarly, internal rotation loading resulted in the lowest stress at the medial first screw site in T3S (64.86 MPa), compared to significantly higher stresses in T2S (136.29 MPa) and T2SBS2 (158.11 MPa). The T2SBS2 configuration showed effective stress reduction at the distal interlocking screws, highlighting its potential for improved load sharing. **Conclusions**: The findings underscore the importance of the secure fixation of the initial interlocking screw in distal tibial fractures. The fixation model utilizing three interlocking screws provided the most favorable overall stress distribution, whereas the inclusion of blocking screws effectively reduced stress concentrations at distal screw sites.

## 1. Introduction

The operative treatment of distal tibial fractures presents distinct challenges due to the anatomical features and biomechanical demands of the lower extremity [1]. The distal tibial canal’s widening, coupled with the presence of fragile cancellous bone, adds complexity to the fixation process, particularly when considering the load-bearing nature of the tibia [2]. Operative treatments regarding intramedullary nailing (IMN) and minimally invasive plate osteosynthesis have demonstrated favorable outcomes in managing these fractures [3]. However, the tibia’s subcutaneous location makes it more prone to open fractures compared to other bones, and severe open fractures are often associated with a high rate of complications and poor long-term results. The elevated risk of postoperative soft tissue complications also makes IMN more favorable for treatment in these cases. Despite concerns regarding the potential for toggling effects and malunion following IMN for distal tibial metadiaphyseal fractures, the advent of blocking screws has significantly improved the ability to achieve better reduction and stable fixation [4]. While blocking screws serve as a valuable tool for reduction, there remains a lack of discussion on their influence on stress distribution around the fixation site.

Finite element (FE) analysis has emerged as a valuable tool in orthopedic biomechanics, offering detailed insights into the stress and strain experienced by bone–implant constructs [5]. Compared to traditional biomechanical testing, FE analysis provides a more comprehensive understanding of how different fixation strategies perform under various load conditions.

The potential benefits of incorporating blocking screws into interlocking screw systems in intramedullary nailing have not been thoroughly investigated. The aim of this study is to assess the stress distribution and structural stability of fixation models incorporating blocking screws under both axial and rotational loading conditions. This analysis is expected to provide meaningful insights into the clinical relevance of blocking screws in distal tibial nailing. It is hypothesized that incorporating blocking screws into the interlocking screw system will not alter the stress distribution or the overall structural integrity of the fixation constructs.

## 2. Materials and Methods

### 2.1. Construction of the Finite Element Model

Institutional Review Board approval was not required for this study, as it was based entirely on a commercially available three-dimensional (3D) CAD dataset of a fourth-generation synthetic left tibia model (SAWBONES^®^, Vashon, WA, USA) [6]. Using SolidWorks 2019^®^ (Dassault Systèmes, Waltham, MA, USA), a full-scale 3D representation of a distal tibial intramedullary fixation system, including a Zimmer^®^ Natural Nail^®^ (Zimmer, Inc., Warsaw, IN, USA), was constructed. Both the tibial geometry and implant designs were processed and refined within the SolidWorks platform, then meshed with 2.0 mm tetrahedral elements to prepare for finite element (FE) analysis. A uniform mesh size of 2 mm was specified for all tetrahedral elements to optimize the balance between numerical accuracy and computational time, based on convergence criteria demonstrating stable stress predictions with this element size [7].

**Figure 1:**
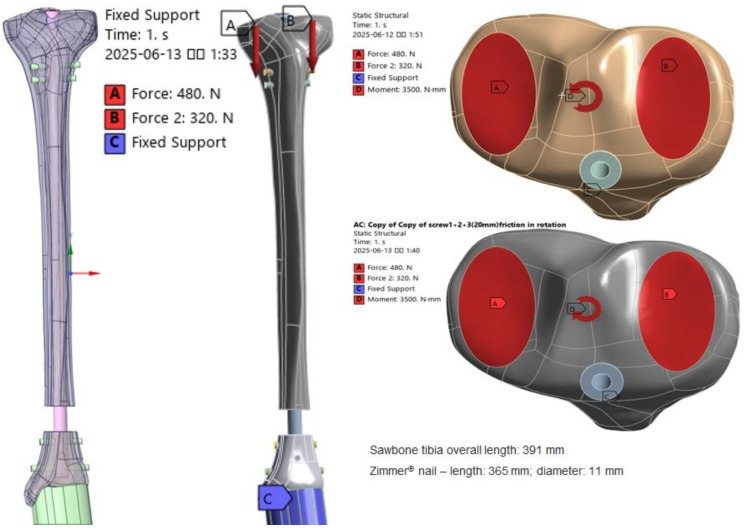
Structural overview of the finite element model based on the fixation configuration. The implant–bone interfaces were modeled as fully bonded proximally, with no-separation and frictional contacts distally, to replicate physiological conditions more realistically.

To simulate a metaphyseal fracture, the models were configured to reflect the anatomy of the distal tibia, with a 20 mm bone gap introduced to avoid cortical contact and to ensure that load transmission occurred exclusively through the nail and screw assemblies from the proximal tibia to the distal fixation components. Fracture patterns were modeled using the same CAD software (SolidWorks 2019) and then exported to ANSYS^®^ software (version 19.0; ANSYS Inc., Canonsburg, PA, USA), where implant positioning was fine-tuned and the final mesh for FE analysis was generated. The construction of the FE models followed these principles: screw placement was determined by the pre-set trajectory of the locking hole angles; the contact between the screws and the plate simulated interlocking fixation behavior; and each screw was advanced to engage the far cortex. Three nail fixation configurations were analyzed: Model A (T2S) used two transverse interlocking screws at the distal segment; Model B (T3S) incorporated three interlocking screws in total, with an additional anteroposterior (AP) screw at the midpoint; and Model C (T2SBS2) consisted of two transverse interlocking screws along with two blocking screws inserted between the interlocking screws (Figure 1).

### 2.2. Modeling of Mechanical Properties and Boundary Conditions with Stress Distribution Analysis

Material characteristics for the synthetic tibia were defined based on the fourth-generation Sawbones specifications provided by the manufacturer (Table 1). Cortical bone was modeled with a Young’s modulus of 10.7 GPa and a Poisson’s ratio of 0.33, while cancellous bone was assigned a lower stiffness value, with a Young’s modulus of 0.524 GPa and a Poisson’s ratio of 0.35. The densities were set at 1.5 g/cm^3^ for cortical bone and 0.2 g/cm^3^ for cancellous bone, in accordance with values previously reported in the literature. All implant components were assumed to be composed of titanium alloy with linear elastic, homogeneous, and isotropic material properties. The titanium alloy was defined by a Young’s modulus of 110 GPa, a Poisson’s ratio of 0.30, and a density of 4.62 g/cm^3^ [8,9,10].

The contact interactions between the implant and bone were defined according to the mechanical characteristics of each interface. A bonded contact was applied between the proximal tibia and the nail, as well as between the proximal tibia and the interlocking screws. In these regions, the bonded setting assumes no sliding or separation, mimicking a glued interface that maintains constant contact throughout loading and allows for a linear solution. The contact between the nail and screws, as well as between the distal tibia and the nail, was defined as no separation. This setting prevents separation between the contacting surfaces while still allowing relative sliding, thus reflecting a more physiologically realistic condition compared to fully bonded contact. The interface between the distal tibia and the screws was defined as frictional contact with a coefficient of friction of 0.2. This setting allows for shear stress transfer up to a defined threshold, after which sliding occurs, thereby capturing the partial resistance to motion typically seen at bone–screw interfaces in the distal metaphysis. The tibiotalar joint was modeled using a bonded contact condition.

**Figure 2:**
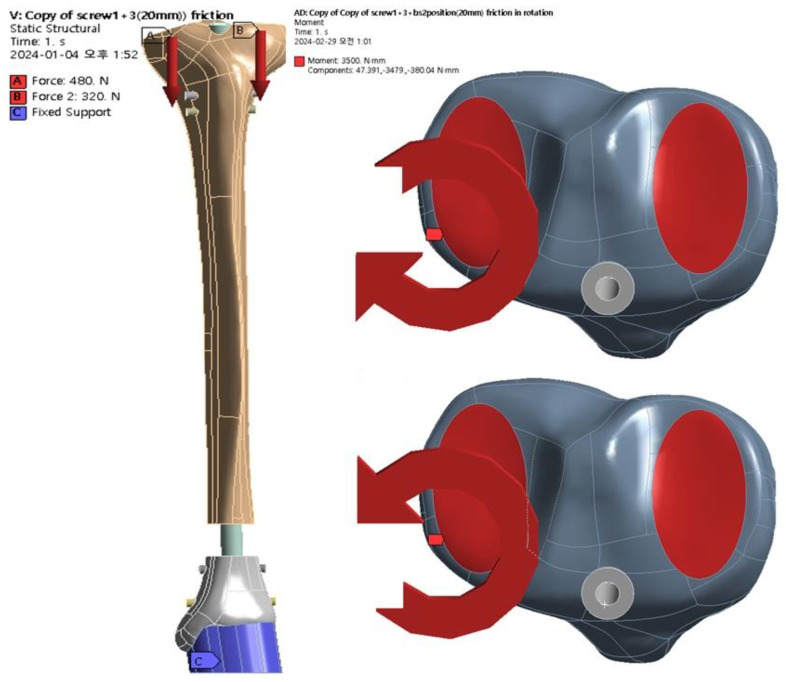
Illustration of static loading conditions applied to the FE models. The axial load at the proximal tibia was distributed asymmetrically in a medial-to-lateral ratio of 3:2. Internal and external rotational loads were simulated around the medial condyle axis of the proximal tibia.

Three loading conditions were simulated to evaluate the mechanical behavior of the fixation constructs under conditions intended to approximate physiological loading as possible. Specifically, three distinct loading modes were considered: static axial loading, internal rotation, and external rotation about the medial condyle axis. For the static loading condition, an axial load of 800 N was applied along the longitudinal axis of the tibia, distributed as 480 N medially and 320 N laterally to approximate an in vivo 55/45 medial–lateral load split (Figure 2) [11]. A torsional moment of 3500 N·mm was applied about the medial condyle axis to replicate in vivo tibial torsion during high-flexion activities [12]. All implant components were modeled as linear elastic, homogeneous, and isotropic materials. Given that the applied loads (800 N axial, 3500 N·mm torsional) are well below the yield strength of Ti-6Al-4V alloy (≈880 MPa), the linear elastic assumption is valid and enables pure comparison of geometric and boundary condition effects on stress distribution. These torsional loads were simulated around the medial condyle of the proximal tibia, which served as the rotational axis. Binding contact was defined between the internal fixation screws and the tibia in accordance with previously validated finite element testing protocols [13]. Finite element analysis was performed using commercially available Ansys^®^ software, and the outcomes were assessed in terms of von Mises stress (VMS), measured in megapascals, and relative displacement of the implant with respect to the bone, which served as an indicator of fixation stability.

## 3. Results

**Figure 3:**
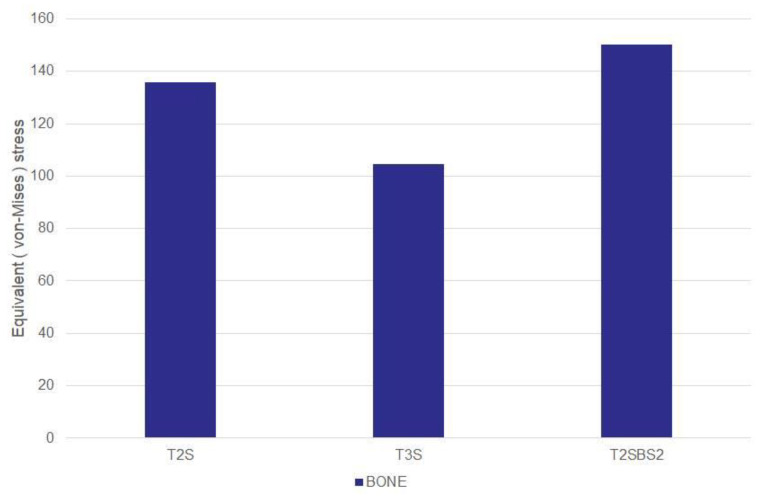
Comparison of maximum von Mises stress (MPa) in the distal tibia among different fixation models. The lowest peak stress was observed in the T3S model.

The maximum VMS in the distal tibia varied by fixation method (Figure 3). The VMS was lowest in the T3S model with three interlocking screws, indicating a more favorable distribution. In contrast, the T2SBS2 model with additional blocking screws showed the highest stress, suggesting that blocking screws may increase local contact stress.

**Figure 4:**
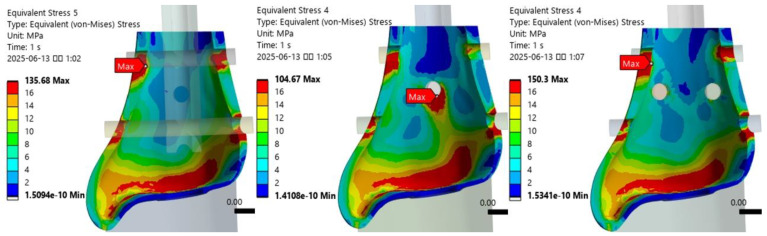
Locations of peak stress at the bone–screw interfaces under axial loading conditions for each fixation model.

Under axial loading conditions, the locations of peak stress at the bone–screw interface were identified for each fixation model (Table 2). In the T2S model, the highest stress was observed at the medial entry point of the first interlocking screw. In the T3S model, it occurred at the anterior entry point of the second anteroposterior screw, while in the T2SBS2 model, peak stress was again noted at the medial insertion site of the first screw (Figure 4). The VMS at the medial contact of the first interlocking screw was lowest in the T3S model, measuring 48.6% of the value observed in the T2S model. Following the addition of blocking screws, the stress distribution across the medial and lateral contacts of the first and third interlocking screws was altered. In particular, the T2SBS2 model showed a reduction in VMS at the medial and lateral contact areas of the third interlocking screw by approximately 20.7% and 27.0%, respectively, compared to the T2S model.

**Figure 5:**
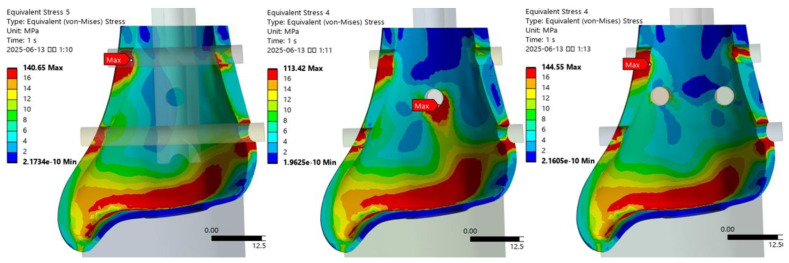
Stress distribution patterns at the bone–screw interfaces under internal rotation loading conditions across fixation models.

**Figure 6:**
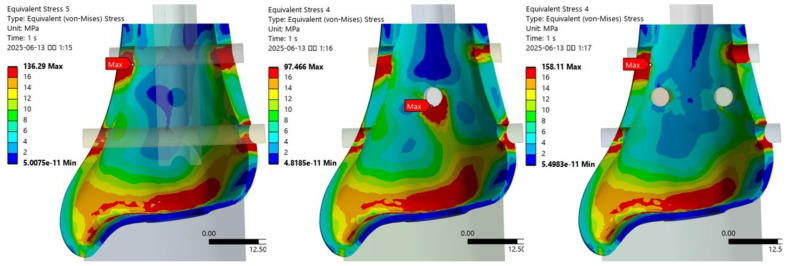
Stress distribution patterns at the bone–screw interfaces under external rotation loading conditions across fixation models.

The stress distribution patterns across the fixation models under internal rotation loading were generally consistent with those observed under axial loading (Figure 5). In the T2SBS2 model, stress was redistributed between the first and third interlocking screws (Table 3). The VMS at the medial contact of the first screw increased from 140.7 MPa in the T2S model to 144.6 MPa in the T2SBS2 model, reflecting a 2.8% increase. In contrast, the medial contact stress at the third screw decreased from 47.8 MPa to 39.3 MPa (17.8% reduction), and the lateral contact stress at the same screw dropped from 60.2 MPa to 50.3 MPa (16.5% reduction). Under external rotation loading, a similar redistribution of stress was observed in the T2SBS2 model (Figure 6, Table 4). The medial contact stress at the first interlocking screw increased from 136.3 MPa in the T2S model to 158.1 MPa in the T2SBS2 model (16.0% increase). Meanwhile, the medial and lateral stresses at the third screw decreased from 45.3 MPa to 37.5 MPa (17.2% reduction) and from 62.0 MPa to 40.1 MPa (35.3% reduction), respectively.

## 4. Discussion

This study investigated stress distribution patterns in various distal tibial intramedullary nail fixation configurations using finite element analysis. Our results revealed distinct differences among the three fixation models. The fixation model employing three interlocking screws demonstrated the lowest maximum von Mises stress (VMS) in the distal tibia, indicating improved load distribution. Importantly, the addition of blocking screws altered stress distribution between interlocking screws under axial loading, resulting in approximately 20.7% and 27.0% reductions in VMS at the medial and lateral contact areas of the distal interlocking screw, respectively. Similar patterns of stress redistribution were observed under internal and external rotational loading conditions.

Intramedullary nailing has evolved considerably since its introduction by Gerhard Küntscher in the 1940s [14]. While offering advantages like minimal incisions and preservation of periosteal blood supply, IMN application to distal tibial fractures presented specific challenges due to metaphyseal widening and thin cortices, often resulting in malalignment and instability [15]. Blocking screws (poller screws) were introduced by Krettek et al. in the late 1990s to improve reduction and fixation stability in metaphyseal fractures [4,16]. These screws narrow the medullary canal, prevent nail translation, and guide the nail centrally through the distal fragment, thereby augmenting mechanical stability by increasing contact points between nail and bone [17,18]. Numerous clinical studies have demonstrated their efficacy in achieving and maintaining proper alignment in distal tibial fractures [19]. Nonetheless, while the role and utility of blocking screws in fracture management have been well documented, few studies have specifically analyzed their impact on stress redistribution within the bone–implant construct. This study reveals that blocking screws alter stress distribution patterns around interlocking screws across all loading conditions tested.

Our findings indicate that the first interlocking screw plays a crucial role in overall construct stability. In both T2S and T2SBS2 models, the highest stress concentrations occurred at this screw’s medial entry point. The addition of blocking screws slightly increased stress at this location under rotational loading conditions, suggesting that accurate placement and secure fixation of the first interlocking screw are paramount, particularly when incorporating blocking screws. Also, the stress redistribution observed in our model demonstrates that blocking screws fundamentally alter the load transfer mechanism through the bone–implant interface. By creating additional contact points between the nail and surrounding bone, blocking screws effectively redirect forces away from the most distal interlocking screw. The stress redistribution achieved by blocking screws may reduce the required rigidity of the fixation construct, potentially minimizing the need for rigid locking plates in comminuted distal metaphyseal fractures. Despite certain considerations including additional surgical steps, radiation exposure, and the need for precise positioning, the biomechanical advantages demonstrated suggest that blocking screws represent a valuable adjunct to standard IMN for distal tibial fractures. The stress redistribution effect may provide mechanistic explanations for clinical success reported in previous studies [20,21].

This study has several limitations. First, the fourth-generation Sawbones synthetic bone model used in this study provides high reproducibility and anatomical accuracy through an injection-molding process that consistently replicates anatomical geometry and cortical thickness [6]. However, it may not fully capture the variability in bone density and microarchitecture observed among human specimens. Therefore, the stress distribution patterns reported here should be interpreted with caution, considering these modeling simplifications. Additionally, the loading conditions applied in this analysis were limited to static loading without incorporating fatigue analysis; consequently, our findings offer fragmentary insights primarily focused on stress distribution and displacement under maximal load conditions. Another potential limitation is that ankle valgus or varus stress testing at the distal fragment was not included. The tibiotalocalcaneal joint has complex interactions across three anatomical planes, meaning internal rotation applied proximally at the tibia can induce external rotation of the hindfoot, generating a valgus force at the ankle joint. Simplifying loading conditions to single-plane simulations might thus deviate from the intended realistic modeling of clinical biomechanics. Future studies incorporating fatigue loading, realistic bone properties, multi-plane stress conditions at the ankle, and direct measurements of interfragmentary movement will help to elucidate both the stress environment and fracture-healing mechanisms in more fracture-specific models.

The clinical implications of our findings extend to surgical decision-making. Surgeons should prioritize accurate placement and secure fixation of the first interlocking screw when incorporating blocking screws. For metaphyseal fractures, our results suggest that a less rigid fixation approach at the most distal aspect may be sufficient with appropriately utilized blocking screws, potentially reducing surgical complexity without compromising stability.

## 5. Conclusions

This finite element analysis shows that blocking screws in distal tibial intramedullary nailing alter stress distribution at the bone–implant interface. Specifically, the three-interlocking-screw model exhibited the lowest peak von Mises stresses under axial, internal rotation, and external rotation loading, and the addition of blocking screws reduced distal screw stress concentrations by approximately 20–27%. This redistribution decreases stress concentration distally, emphasizing the importance of accurate fixation of the first interlocking screw and allowing less rigid fixation at the metaphysis. These findings clarify the biomechanical role of blocking screws, potentially guiding more effective distal tibial fracture fixation strategies.

## Figures and Tables

**Table 1:** Material properties of bone and implant.

Materials	Density (g/cm^3^)	Elastic Modulus (GPa)	Poisson’s Ratio
Cortical bone	1.5	10.7	0.33
Cancellous bone	0.2	0.524	0.35
Titanium alloy implant	4.62	110	0.30

**Table 2:** Stress distribution under axial loading conditions for each fixation models.

Bone–Implant Interface	T2S	T3S	T2SBS2
1st interlocking screw medial contact	135.7	66.0	150.3
1st interlocking screw lateral contact	37.9	17.5	44.3
2nd interlocking screw anterior contact		104.7	
3rd interlocking screw medial contact	48.6	43.2	38.5
3rd interlocking screw lateral contact	57.6	45.2	42.0

**Table 3:** Stress distribution under internal rotation loading conditions for each fixation models.

Bone–Implant Interface	T2S	T3S	T2SBS2
1st interlocking screw medial contact	140.7	74.0	144.6
1st interlocking screw lateral contact	23.2	14.0	31.3
2nd interlocking screw anterior contact		113.4	
3rd interlocking screw medial contact	47.8	34.1	39.3
3rd interlocking screw lateral contact	60.2	43.0	50.3

**Table 4:** Stress distribution under external rotation loading conditions for each fixation models.

Bone–Implant Interface	T2S	T3S	T2SBS2
1st interlocking screw medial contact	136.3	64.9	158.1
1st interlocking screw lateral contact	63.5	26.5	58.0
2nd interlocking screw anterior contact		97.5	
3rd interlocking screw medial contact	45.3	42.2	37.5
3rd interlocking screw lateral contact	62.0	48.6	40.1

## Data Availability

The datasets generated and analyzed during the current study are available from the corresponding author upon reasonable request.

## Abbreviations

The following abbreviations are used in this manuscript:

T2S	Finite element models with two interlocking screws configuration
T3S	Finite element models with three interlocking screws configuration
T2SBS2	Finite element models with two interlocking screws with two blocking screws
VMS	von Mises stress
